# Porf-2 Inhibits Tumor Cell Migration Through the MMP-2/9 Signaling Pathway in Neuroblastoma and Glioma

**DOI:** 10.3389/fonc.2020.00975

**Published:** 2020-06-26

**Authors:** Xue-Yuan Li, Guo-Hui Huang, Qian-Kun Liu, Xi-Tao Yang, Kang Wang, Wen-Zheng Luo, Tian-Song Liang, Shan-Peng Yuan, Ying-Wei Zhen, Dong-Ming Yan

**Affiliations:** ^1^Department of Neurosurgery, The First Affiliated Hospital of Zhengzhou University, Zhengzhou, China; ^2^Department of Otolaryngology-Head and Neck Surgery, Shanghai Ninth People's Hospital, Shanghai Jiao Tong University School of Medicine, Shanghai, China; ^3^Ear Institute, Shanghai Jiao Tong University School of Medicine, Shanghai, China; ^4^Shanghai Key Laboratory of Translational Medicine on Ear and Nose Diseases, Shanghai, China; ^5^Department of Interventional Therapy, Shanghai Ninth People's Hospital, Shanghai Jiao Tong University School of Medicine, Shanghai, China

**Keywords:** Porf-2, tumor migration, MMP-2/9, neuroblastoma, glioma

## Abstract

Tumor migration and invasion are key pathological processes that contribute to cell metastasis as well as treatment failure in patients with malignant tumors. However, the mechanisms governing tumor cell migration remain poorly understood. By analyzing the tumor-related database and tumor cell lines, we found that preoptic regulatory factor-2 (Porf-2) is downexpressed in both neuroblastoma and glioma. Using *in vitro* assays, our data demonstrated that the expression of Porf-2 inhibits tumor cell migration both in neuroblastoma and glioma cell lines. Domain-mutated Porf-2 plasmids were then constructed, and it was found that the GAP domain, which plays a role in the inactivation of Rac1, is the functional domain for inhibiting tumor cell migration. Furthermore, by screening potential downstream effectors, we found that Porf-2 can reduce MMP-2 and MMP-9 expression. Overexpression of MMP-2 blocked the inhibitory effect of Porf-2 in tumor cell migration both *in vitro* and *in vivo*. Taken together, we show for the first time that Porf-2 is capable of suppressing tumor cell migration via its GAP domain and the downregulation of MMP-2/9, suggesting that targeting Porf-2 could be a promising therapeutic strategy for nervous system tumors.

## Introduction

Tumor migration and invasion are a hallmark of most human cancers and remain a major source of treatment failure in patients with malignant tumors (1, 2). Nervous system tumors represent some of the most aggressive cancers in both children and adults, particularly neuroblastoma and glioblastoma multiforme. Despite recent advances in standard therapy, including surgical resection followed by radiation and chemotherapy, the prognosis for patients with malignant glioma and neuroblastoma remains dismal due to the highly proliferative, migratory, and invasive capacity of tumor cells (3–8). Although numerous studies have attempted to elucidate the molecular mechanisms of tumor migration in recent years, the results are not satisfactory. Additionally, there are currently no clinically used therapies that effectively slow the spread of tumor cells. The mechanisms governing tumor migration remains elusive, and the discovery of new potential target oncogenes or tumor suppressor genes for anticancer drug development remains a major goal for cancer research (1, 2). Thus, there is an urgent need to investigate the molecular mechanisms underpinning tumor cell migration.

Many studies have identified the key role of Rho GTPase activity and its mediators (RhoGAPs, RhoGEFs, RhoGDIs) in regulating tumor migration and invasion (9, 10). Preoptic regulatory factor-2 (Porf-2), also known as Cross GTPase-activating protein (CrossGAP)/Vilse, belongs to the Rho GAP family and was first discovered in the preoptic area of the hypothalamus in castrated male rats (11). Previous studies mainly focus on the role of Porf-2 in axon guidance and dendritic spine formation during the development of the central nervous system (12–15). As previously reported, Porf-2 plays a pivotal role in neural stem cell proliferation and axon outgrowth (16–18). However, despite this, there is no data on the effect of Porf-2 on the behavior of tumor cells. Porf-2 has three domains: the WW domain, Myosin Tail Homology 4 (Myth4) domain, and GTPase activating proteins (GAP) domain, but, up to now, the function of these three domains is rarely reported.

Herein, by analyzing the Cancer Genome Atlas (TCGA) database and tumor cell lines, we found that Porf-2 is underexpressed in glioma and neuroblastoma. Furthermore, the overexpression of Porf-2 inhibited tumor cell migration in both the neuroblastoma cell line (Neuro-2a) and the glioma cell line (U87). Knockdown of Porf-2 expression promoted tumor cell migration, and it was found that the GAP domain contributed to this phenotype. By screening potential downstream effectors, we found that matrix metalloproteinase-2/9 (MMP-2/9) expression was decreased after Porf-2 overexpression. Furthermore, overexpression of MMP-2 negated the effect of Porf-2 in tumor cell migration. Taken together, we show for the first time that Porf-2 inhibits tumor cell migration through the MMP pathway via its GAP domain. This data suggests that Porf-2 may function as a potential tumor suppressor, which provides new insight on clinical targets for tumor cell migration in the nervous system.

## Materials and Methods

### Cell Culture

Glioma cell line U87 and U251, and neuroblastoma cell line Neuro-2a and SK-N-SH, SH-SY5Y, were obtained from the China Academia Sinica Cell Repository (Shanghai, China). The cells were cultured in Dulbecco's modified Eagles medium (DMEM; Gibco) supplemented with 10% fetal bovine serum (FBS; Gibco) and incubated at 37°C in a 5% CO2 and 95% humidified air atmosphere. The neuronal cell line NG108 was cultured in DMEM supplemented with 10% FBS, 1% penicillin-streptomycin, and 0.1 mM hypoxanthine.

### Quantitative Real-Time PCR

Total RNA was extracted from cultured cells using TRIzol reagent (Invitrogen) according to the manufacturer's instructions. Gene expression was quantified by quantitative real-time (qRT)–PCR using the SYBR Green PCR Master Mix (Applied Biosystems, 4385612) and an Applied Biosystems 7,500 Fast Real-Time PCR system. PCR was performed with the corresponding primers (Supplemental Table 1). Individual samples were run in triplicate, and each experiment was repeated at least three times. Data analyses were performed using the comparative CT method for calculating the relative gene expression.

### Wound Healing Assay

A scratch was made in the middle of the well with a pipette tip 24 h after transfection, and the monolayer was washed twice with PBS. The cells were cultured in serum-free media and incubated in a 5% CO_2_ and 95% humidified air atmosphere at 37°C for 96 h. Six randomly selected fields at the lesion border were then photographed using an inverted microscope at the indicated time. The wound area (the area without cells) was measured during the period and was normalized to each group at 0 h. Experiments were carried out in triplicate and repeated at least three times.

### Western Blot Analysis

At the designated time, the cells were washed twice with PBS, homogenized, and lysed in cell lysis buffer (Beyotime, P0013) supplemented with 1 mM PMSF, 50 mM NaF, 1 mM Na_3_VO_4_, and protease inhibitor; western blot analysis was then performed as described (17). Briefly, the total protein lysates were separated by SDS-PAGE and analyzed by western blotting with anti-GAPDH (1:5000, Sigma-Aldrich, G8795), anti-β-actin (1:5000, Thermofisher scientific, MA5-15739), anti-HA (1:1000, Cell Signaling Technology, 2367s), anti-Porf-2 (1:1000, Santa Cruz Biotechnology, 87186), anti-MMP-2 (1:1000, Cell Signaling Technology, 87809), and anti-MMP-9 (1:1000, Abcam, ab38898). HRP-conjugated anti-rabbit, anti-mouse, and anti-goat secondary antibodies (Beyotime, A0208, A0216, and A0181) were also used. Analysis of the data was performed using NIH ImageJ software. The mean density of each band was normalized to the actin or GAPDH signal in the same sample.

### CCK8 Proliferation Assay

The proliferation of cells was determined using a CCK-8 assay, which is a sensitive, non-radioactive colorimetric assay (Beyotime, C0037). The cells were dissociated mechanically and seeded into 96-well plates (5 × 10^3^ cells/well) and were cultured in growth medium for different periods (0, 24, 48, 72, or 96 h). CCK-8 solution was added to the cell culture medium to a final concentration of 10 μl per 100 μl of medium and incubated at 37°C in a 5% CO_2_ air atmosphere. Absorbance was measured 4 h later at a wavelength of 450 nm using a microplate reader (BioTek).

### Trans-well Assay

For cell migration and invasion assays, a total of 5 × 10^4^ transfected cells were resuspended in 200 μl of serum-free medium and placed in the upper compartment of a Trans-well chamber (Corning; 24-well insert, pore size: 8 μm). The lower chamber was filled with 15% FBS as a chemoattractant and incubated for 24 h for the migration assay. For the invasion assay, the poly-carbohydrate membrane was coated with Matrigel basement membrane matrix. After 24 h of incubation, the upper side of the filter was scraped with a cotton tip to eliminate cells that had not migrated through it. At the end of the experiments, the cells on the upper surface of the membrane were removed, and the cells on the lower surface were fixed with methanol and stained with crystal violet (Beyotime, C0121). Six visual fields of each insert were randomly chosen and counted under a light microscope. Each experiment was repeated at least three times.

### Expression Plasmids and Cell Transfection

Expression plasmids for wild-type and mutant Porf-2 were constructed as reported (17). Briefly, the cDNA fragment encoding Porf-2 was PCR-amplified using mouse hippocampus cDNA as a template. The fragment was inserted into a plvx-IRES-zsGreen plasmid. The mutant Porf-2 was also PCR-amplified by mutant primers and inserted into a plvx-IRES-zsGreen vector. Transient transfection was performed with U87 and Neuro-2a (N-2a) cells using Lipofectamine 2,000, according to the manufacturer's instructions. Cells transfected with empty plasmid vectors were used as controls. The sequence of Porf-2 shRNAs was constructed as previously reported and validated (17). All shRNAs were also inserted into pLKO.1 vectors, as reported (17).

### Rac1/Cdc42 Activation Assay

Rac1/Cdc42 activity was assessed using the Rac1/Cdc42 Activation Assay Kit (Millipore) according to the manufacturer's instructions. Briefly, cell lysates were clarified by centrifugation at 14,000 × g at 4°C for 10 min. Equal volumes of lysates were incubated with beads to pull down activated Rac1 proteins. After incubation at 4°C for 1 h, the beads were washed three times with cold MLB buffer. The Rac1 proteins were eluted with sample buffer and subjected to sodium dodecyl sulfate-polyacrylamide gel electrophoresis (SDS-PAGE). Western blot analysis was performed using anti-Rac1 or Cdc42 antibodies (Millipore).

### Tumor Xenografts in Nude Mice

Five-week-old, 18–22 g, athymic male BALB/c nude mice were raised in specific pathogen-free conditions. The mice were implanted U87 or N-2a cells transfected with the Control, Porf-2, Porf-2+MMP2, respectively. The cells (about 5 × 10^6^) were injected subcutaneously into the flank of the final volume of 100 μL. Mice were killed 28 days after initial injection of tumor cells. The length and the width of the visible tumors were measured, with the volume of the tumors to be calculated according to the formula V=(length × width^2^)/2. The mice that died or did not grow tumors, or developed skin ulcers during the experiment were not included in the statistical analysis. The animal experiments were approved by the Ethics Committee of Zhengzhou University and performed in accordance with the guidelines of Laboratory Animal Care (National Society for Medical Research).

### Statistical Analysis

All experiments were performed at least three times in triplicate. All values are expressed as mean ± SEM. The statistically significant differences between two groups were evaluated using the Student's *t*-test. All data analyses were performed using the SPSS Software. ANOVA and Tukey's multiple comparison tests were used for the analysis of differences in three or more groups. Differences were considered statistically significant at *P* < 0.05. ^*^ *P* < 0.05, ^**^ *P* < 0.01, ^***^ *P* < 0.001.

## Results

### Porf-2 Expression Is Decreased in Both Neuroblastoma and Glioma Cell Lines

**Figure 1:**
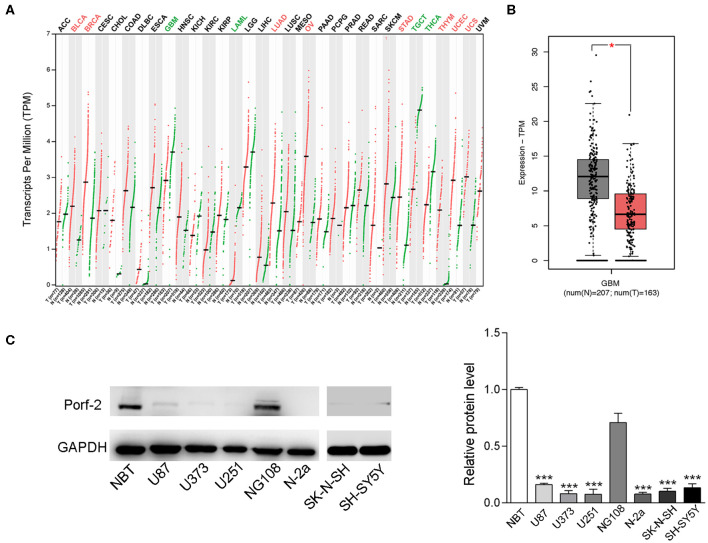
Porf-2 expression is decreased in both neuroblastoma and glioma cell lines. **(A)** Porf-2 expression levels were analyzed in several tumors from the TCGA database. All the abbreviations of the cancer name can be found on the TCGA website. https://gdc.cancer.gov/resources-tcga-users/tcga-code-tables/tcga-study-abbreviations.The cancer names in black indicate that there is no differential expression between the cancer type tumor and its adjacent or normal tissue, red indicates that it is upregulated in the tumor tissue, and green indicates that it is downregulated in the tumor tissue. On the horizontal axis, T represents the tumor, n represents the control, the number n in parenthesis represents the corresponding sample number. **(B)** The box plot of the TCGA database indicates that Porf-2 expression is downregulated in glioma. **(C)** The expression of Porf-2 in neuroblastoma and glioma cell lines were detected and quantified by western blotting. NBT represents normal brain tissue. Error bars represent ± SEM; ^*^P < 0.05, ^***^P < 0.001.

To investigate the impact of Porf-2 in tumors, especially in nervous system tumors, we analyzed the gene expression profiling data from the TCGA database. Our analysis revealed that Porf-2 is downregulated in glioma, acute myeloid leukemia, testicular germ cell tumors, and thyroid carcinoma, while it is unregulated in bladder urothelial carcinoma, breast invasive carcinoma and several other tumors (Figure 1A). The box plot after analysis of the TCGA database also showed that Porf-2 is significantly downexpressed in glioma (Figure 1B). As Porf-2 is reported to be highly expressed in the nervous system, such as brain and ganglion (14), we focused on its involvement in nervous system tumors, especially glioma and neuroblastoma. We further assessed the expression of Porf-2 in glioma and neuroblastoma cell lines. We found that Porf-2 is downexpressed in both glioma cell lines U-87 MG, U-373 MG, and U-251, as well as neuroblastoma cell line Neuro-2a (N-2a), SK-N-SH and SH-SY5Y (Figure 1C), suggesting that Porf-2 potentially plays a similar role in glioma and neuroblastoma.

### Porf-2 Inhibits Neuroblastoma and Glioma Cell Migration

**Figure 2:**
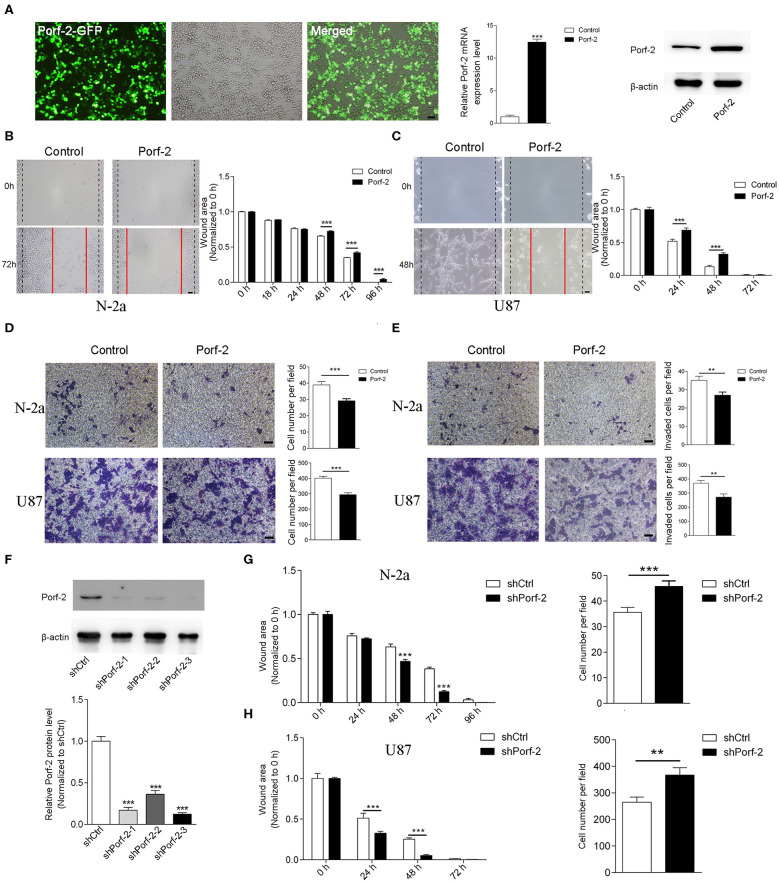
Porf-2 suppressed Neuro-2a cell migration. **(A)** The GFP image shows the overexpression of Porf-2 in N-2a cells. RT-PCR and western blotting confirmed the overexpression of Porf-2. β-actin was used as a loading control. Scale bar, 200 μm. **(B)** Representative images of the wound healing assay of N-2a cells at different time points in the control and Porf-2 group. The red lines indicate the wound area. Scale bar, 200 μm. Quantification of the wound area at the indicated time points in the control and Porf-2 group. **(C)** Representative images of the wound healing assay of U87 cells at different time points in the control and Porf-2 group. The red lines indicate the wound area. Scale bar, 200 μm. **(D)** Representative images of the N-2a and U87 cells that migrated to the lower chamber in the trans-well assay in control and Porf-2 group. Scale bar, 200 μm. Quantification of the migrated cells. **(E)** Representative images of the N-2a and U87 cells that invaded into the lower chamber. Scale bar, 200 μm. Quantification of the invaded cells in control and Porf-2 group. **(F)** Western blots showing Porf-2 knockdown with shRNAs. **(G)** Quantification of the wound area of N-2a cells at the indicated time points in the shCtrl and shPorf-2 groups. Quantification of migrated N-2a cells of the trans-well assay in the shCtrl and shPorf-2 groups. **(H)** Quantification of the wound area of U87 cells at the indicated time points in the shCtrl and shPorf-2 groups. Quantification of migrated U87 cells of the trans-well assay in the shCtrl and shPorf-2 groups. Error bars represent ± SEM; ^**^P < 0.01, ^***^P < 0.001.

Porf-2 is downexpressed in glioma and neuroblastoma cell lines compared to normal tissue (Figure 1C). This gave us insight into what would happen if Porf-2 was re-expressed in tumor cells. Firstly, we overexpressed Porf-2 by transfecting the Porf-2-GFP plasmid into N-2a cells. The GFP expression in fluorescence microscopy indicated that more than 95% of N-2a cells were successfully transfected, and Real-time PCR and western blot analysis confirmed its overexpression (Figure 2A). Next, we used the wound healing assay to detect the effect of Porf-2 re-expression in tumor cell migration. The wound healing assay results showed that the wound area was significantly larger after Porf-2 re-expression compared to control group from 48 to 96 h (Figure 2B), suggesting that Porf-2 re-expression inhibited the migration of N-2a cells. In U87 cells, we also found that Porf-2 overexpression exhibited a larger wound area from 24 to 48 h compared to the control, indicating its inhibitory function on U87 cell migration (Figure 2C). Additionally, a trans-well assay was used to examine the effect of Porf-2 re-expression on N-2a and U87 migration (Figure 2D). Our data showed that significantly fewer N-2a and U87 cells in Porf-2 overexpression group had passed through the polycarbonate membrane after 24 h than that in the control group (Figure 2D). The invasion ability of Porf-2 was also explored (Figure 2E). Our results showed that the invaded cell number was significantly reduced after Porf-2 overexpression in both N-2a and U87 cells (Figure 2E). Taken together, these results indicated the biological importance of Porf-2 on the migratory behavior of N-2a and U87 cells.

We also evaluated tumor cell migration after knockdown of Porf-2 (Figure 2F). We found that knockdown of Porf-2 showed a smaller wound area compared to the control in both N-2a and U87 cells (Figures 2G,H). Similarly, the trans-well assay showed an increase in migrated tumor cells after the knockdown of Porf-2 in the N-2a and U87 cell lines (Figures 2G,H). These results confirm that the knockdown of Porf-2 promotes tumor cell migration.

### The GAP Domain Is Required for Both Glioma and Neuroblastoma Cell Migration

Porf-2 has three domains: WW, Myth4, and GAP. The WW domain is required for the protein-protein binding to mediate axon and dendritic spine formation in neurons (12, 15, 19). Little is reported on the Myth4 domain (20), and the GAP domain is reported to inactivate Rac1/cdc42 to mediate axon repulsion during neuron development (12, 15, 21). However, the Porf-2 domain responsible for cancer cell migration is unknown.

**Figure 3:**
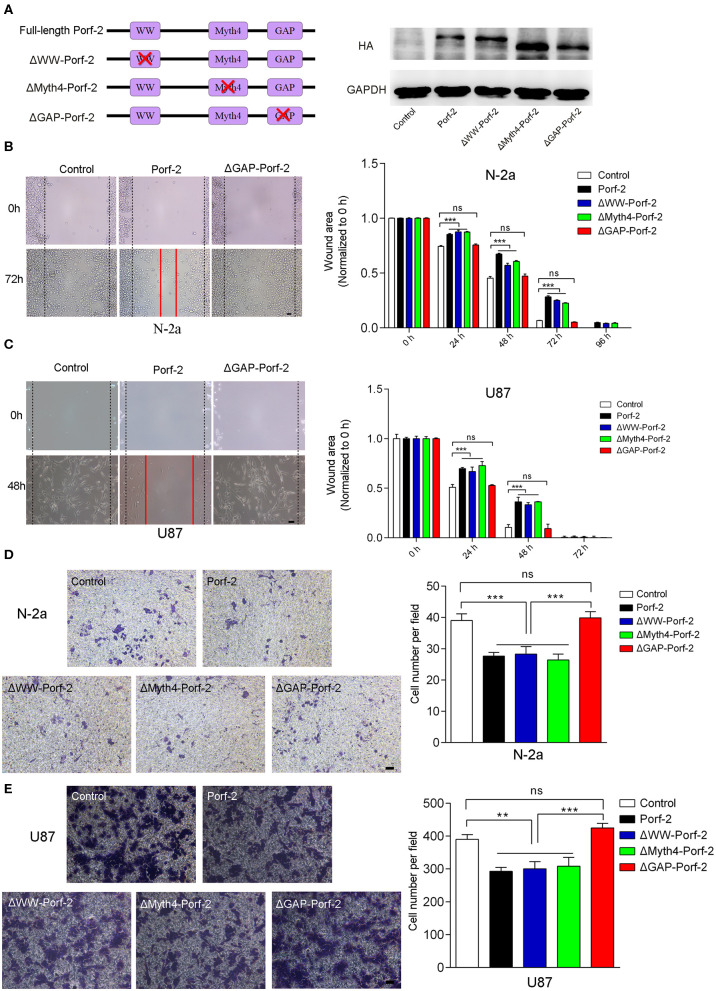
The GAP domain is required for both glioma and neuroblastoma cell migration. **(A)** The construct scheme of the domain-deleted plasmids of Porf-2. Verification of domain-deleted plasmids via western blotting with the HA antibody. The same blot was probed with GAPDH antibody as a loading control. **(B)** Representative images of the wound healing assay of N-2a cells at different time points in different groups. Quantification of the wound area at indicated time points in different groups. Scale bar, 200 μm. **(C)** Representative images of the wound healing assay of U87 cells at different time points in different groups. Scale bar, 200 μm. **(D)** Representative images of the migrated N-2a cells in different groups. Quantification of the migrated cells in each group. Scale bar, 200 μm. **(E)** Representative images of the migrated U87 cells in different groups. Quantification of the migrated cells in each group. Scale bar, 200 μm. Error bars represent ± SEM; ns indicates no significant difference (*P* > 0.05). ^**^P < 0.01, ^***^P < 0.001.

Since there are many different cancer cell types, a factor or protein may play a different role through different domains or mechanisms for each cell type (22–24). To determine whether Porf-2 plays a common function through the same domain in other cell lines, we evaluated the mutant Porf-2 in both N-2a and U87 cell lines. Initially, we constructed the domain-deleted Porf-2 plasmids, and the expression of the mutants was confirmed by western blotting (Figure 3A). We then used the wound healing assay to screen for the domain that affects cancer cell migration. In both N-2a and U87 cells, we found that both ΔWW-Porf-2 and ΔMyth4-Porf-2 played a similar role in the inhibition of cell migration as the unmutated Porf-2, and exhibited a larger wound area than the control (Figures 3B,C). However, the ΔGAP-Porf-2 exhibited no inhibitory function on tumor cell migration, which was similar to that of the control (Figures 3B,C). In other words, the GAP domain is required for Porf-2 to inhibit tumor migration. The trans-well assay further validated this finding. As shown in Figures 3D,E, ΔWW-Porf-2, ΔMyth4-Porf-2, and Porf-2 significantly reduced the migratory cell number of both N-2a and U87 compared to the control group. While ΔGAP-Porf-2 showed no difference compared to the control, suggesting that the GAP domain is responsible for the inhibition of tumor cell migration (Figures 3D,E).

In conclusion, Porf-2 inhibits both glioma and neuroblastoma cell migration, and the GAP domain plays an essential role in this process.

### Porf-2 Inhibits Tumor Cell Migration Through the MMP Signaling Pathway

**Figure 4:**
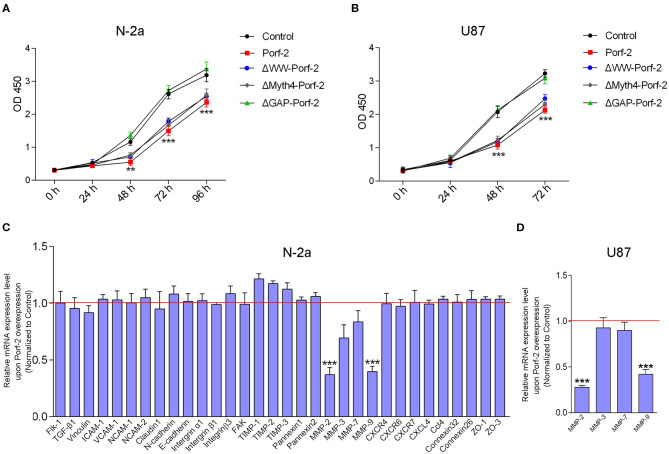
Porf-2 inhibits tumor cell migration through MMPs. **(A)** CCK-8 assays were performed to analyze the proliferation of N-2a cells in different groups. **(B)** CCK-8 assays were performed to assess the proliferation ability of U87 cells in different groups. Error bars represent ± SEM; ^**^P < 0.01, ^***^P < 0.001 vs. the control group. **(C)** Real-time PCR analysis was performed to explore the relative mRNA expression of the potential target genes after Porf-2 overexpression in N-2a. The relative mRNA level was normalized to the control. The red line indicates the normalized control group. **(D)** Real-time PCR was performed to verify the downexpression of MMPs after Porf-2 overexpression in U87 cells. The relative mRNA level was normalized to the control. The red line indicates the normalized control group. Error bars represent ± SEM; ^**^P < 0.01, ^***^P < 0.001 vs. the control group.

As Porf-2 is also reported to associate with stem cell proliferation (18), we explored its potential effect on tumor cell proliferation via the CCK-8 proliferation assay. As shown in Figures 4A,B, we found that overexpression of Porf-2 suppressed N-2a and U87 cell proliferation. Similar to Porf-2, the overexpression of ΔWW-Porf-2 and ΔMyth4-Porf-2 also significantly inhibited cell proliferation compared to the control group (Figures 4A,B). While, after the GAP domain mutation, Porf-2 lost its inhibitory effect on cell proliferation in both N-2a and U87 cells (Figures 4A,B).

**Figure 5:**
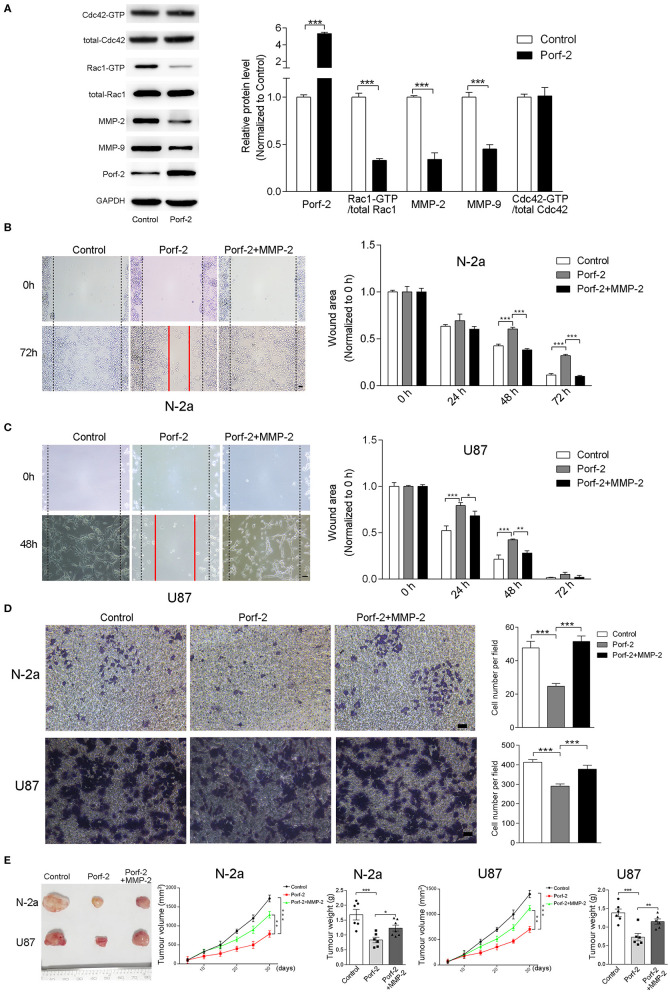
The GAP domain is required for both glioma and neuroblastoma cell migration. The MMP-2/9 and Porf-2 protein levels were detected by western blotting upon Porf-2 overexpression. **(A)** GST pull-down assay was used to detect Rac1/Cdc42 activity upon Porf-2 overexpression. The expression levels of the indicated proteins were quantified. **(B)** Representative N-2a cell images of the wound healing assay at different time points in the control, Porf-2, and Porf-2 + MMP-2 groups. The wound area at the indicated time points in different groups was quantified. Scale bar, 200 μm. **(C)** Representative U87 cell images of the wound healing assay at different time points in the control, Porf-2, and Porf-2 + MMP-2 groups. Scale bar, 200 μm. **(D)** Representative images of the migrated N-2a and U87 cells in the trans-well assay in different groups. Quantification of the migrated cells in each group. Scale bar, 200 μm. **(E)** Isolated tumors derived from tumor xenografts of different groups. Tumor volumes was assessed every 5 days in different groups. Tumor weight was also measured. Error bars represent ± SEM; ^*^P < 0.05, ^**^P < 0.01, ^***^P < 0.001.

It is well-known that tumor cell migration involves multiple processes, such as cell-cell junctions, the formation of membrane protrusions, the plasticity of cell migration, cell-matrix adhesion, and cytoskeletal dynamics, which are associated with several signaling pathways. To understand the possible mechanism of Porf-2 on tumor cell migration, we surveyed several adhesion molecules, chemokines and their receptors, integrins, MMPs, and adhesion-related kinases. As shown in Figure 4C of N-2a cells, Porf-2 overexpression has no effect on E-cadherin, N-cadherin, ICAM1, VCAM1, CXCR4/6/7, CXCL4, intergrinα1/β1/β3, etc. Interestingly, both MMP-2 and MMP-9 expression were significantly reduced upon Porf-2 overexpression in N-2a cells (Figure 4C). We also confirmed the expression of MMP-2 and MMP-9 was down-regulated upon Porf-2 overexpression in U87 cells (Figure 4D). Western blotting also confirmed that Porf-2 overexpression reduces the expression of MMP-2/9 in protein level (Figure 5A).

Considering that the GAP domain was reported to inactive Rac1 or Cdc42, which is involved in tumor cell migration and associated with MMPs, we detected the Rac1 and Cdc42 activity and MMP-2/9 expression level upon Porf-2 expression. We found that Porf-2 overexpression specifically inactivates Rac1 but not Cdc42, and reduces the expression of MMP-2/9 (Figure 5A). To further elucidate the relationship between Porf-2 and the MMPs with regard to the inhibition of tumor cell migration, we designed a rescue assay by overexpression of MMP-2. The over-expression of MMP2 were verified by qRT-PCR and WB (Supplemental Figure 1). As shown in Figures 5B,C, in both N-2a and U87 cells, Porf-2 overexpression exhibited a larger wound area compared to that of control group. However, compared to Porf-2 overexpression, overexpression of MMP-2 exhibited smaller wound area and showed similar genotype as control (Figures 5B,C), suggesting that MMP-2 blocked the effect of Porf-2 on tumor cell migration. Consistently, the trans-well assay indicated that overexpression of MMP-2 abolished the effect of Porf-2 on both N-2a and U87 cell migration (Figure 5D).

Finally, we attempted to confirm the role of Porf-2 and MMP-2 in the N-2a and U87 cells of xenografts *in vivo*. As shown in Figure 5E, compared to control, Porf-2 overexpression significantly reduced the tumor size and weight *in vivo*. Whereas, MMP2 expression can reverse the effect of Porf-2 *in vivo*, showing larger tumor volume and increased tumor weight (Figure 5E).

In general, our results demonstrate that Porf-2 mediates tumor cell proliferation and migration through the MMP signaling pathway.

## Discussion

Our study is the first to demonstrate that Porf-2 is underexpressed both in glioma and neuroblastoma, and also that the overexpression of Porf-2 suppresses cell migration through the MMP-2/9 signaling pathway. Moreover, the GAP domain-deleted Porf-2 had no effect on tumor cell migration. Our findings indicate that Porf-2 inhibited tumor cell migration through the MMP-2/9 signaling pathways via its GAP domain.

The role of Porf-2 in tumors is rarely reported. Previous studies mainly focus on its role in central nervous system development, especially axon outgrowth and guidance, and dendritic spine or synapse formation (12–14, 16, 19). Our knowledge concerning its role in tumorigenesis remains limited. Considering the extensive expression of Porf-2 in the brain and many peripheral tissues, including testes, liver, kidney, prostate, and placenta (14, 25), and its downregulation in several human tumors, Porf-2 may function as a potential tumor suppressor gene in several systems. Herein, we demonstrate that Porf-2 expression is downregulated in nervous system tumors, including glioma and neuroblastoma, and plays an inhibitory role in tumor cell migration.

Migration is a critical process in metastasis. The family of Rho GTPases is a ubiquitously expressed division of GTP-binding proteins involved in the regulation of cytoskeletal dynamics and intracellular signaling (9, 10, 21). Rho GTPase activation is regulated through its association with various RhoGEFs and RhoGAPs, which control the cycle between the GDP- and GTP-bound states (9, 10, 21). In tumors, abnormalities in RhoGAPs and Rho GTPase function have major consequences for cancer progression (9, 10). In this study, we found that Porf-2, which belongs to the RhoGAPs family, participated in tumor cell migration. Previously, it was found that Porf-2 can function as a GAP to inactivate Rac1 or Cdc42 (12, 16, 17, 19). In our current study, we found that the overexpression of Porf-2 reduced the activated Rac1 levels in tumor cells, but not Cdc42, demonstrating that Porf-2 acts as a Rac1-specific GAP in tumor cell migration, which is consistent with previous reports (12, 15). In addition, we identified that the GAP domain is required for the regulation of tumor cell migration. Therefore, as a part of the RhoGAP family, Porf-2 may suppress tumor cell migration through the inactivation of Rac1 via its GAP domain.

Several mechanisms and signaling pathways contribute to tumor migration, invasion, and metastasis, such as cell-cell adhesion, cell-matrix adhesion, cytoskeletal dynamics, and formation of membrane protrusions (1, 2, 26). In the current study, we explored the potential mechanism of Porf-2 in tumor cell migration by screening several key proteins, such as adhesion molecules (ICAM1, VCAM1, E-cadherin), chemokines (CCL4, CXCL4) and their receptors (CXCR4/6/7), integrins (integrin α1/β1/β3), and MMPs (MMP-2/3/7/9). We found that the expression of Porf-2 inhibits the expression of its downstream effectors MMP-2 and MMP-9, the well-known MMPs associated with cell migration and invasion (27). Previous studies revealed that Rac1 activity is involved in the expression of various MMPs (28). We further found that Porf-2 can inactive Rac1 and decrease MMP-2/9 protein level in tumor cells. To elucidate the necessary or the requirement of MMPs in Porf-2's function, we further performed a rescue assay. We found MMP-2 blocked the effect of Porf-2 on tumor cell migration, demonstrating that Porf-2 mediates tumor cell migration through the MMP signaling pathway. Therefore, Porf-2 may inhibit tumor cell migration through the inactivation of Rac1 via the GAP domain, followed by the down-regulation of MMP-2/9. From a therapeutic standpoint, our data suggest that restoring Porf-2 expression could inhibit the migration of tumor cells and prevent it from spreading.

In summary, low expression of Porf-2 was observed in neuroblastoma and glioma. By the overexpression and underexpression of Porf-2, we found that Porf-2 can function as a tumor suppressor gene and inhibit tumor cell migration in both neuroblastoma and glioma via its GAP domain. Furthermore, by screening potential downstream effectors, we found that Porf-2 is able to suppress tumor cell migration by the inactivation of Rac1 and by reducing the expression of MMP-2/9. Overall, these findings grant an understanding of the progression of malignant tumors, which may provide a new target for tumor migration.

## Data Availability Statement

The data used to support the findings of this study are available from the corresponding author upon request.

## Author Contributions

X-YL, Q-KL, and G-HH performed the experiments of cell culture, wound healing, and transwell assay. X-YL, Q-KL, G-HH, and X-TY has been involved in analysis and interpretation of data and revising the manuscript critically. KW, W-ZL, T-SL, S-PY, and Y-WZ supervised the data analysis. X-YL and D-MY designed experiments and revised the manuscript critically. All authors agree that all the questions related to the accuracy or integrity of the paper have been appropriately investigated and resolved, giving final approval of the version to be published.

## Conflict of Interest

The authors declare that the research was conducted in the absence of any commercial or financial relationships that could be construed as a potential conflict of interest.
