# Supplementary material for: Porf-2 Inhibits Tumor Cell Migration Through the MMP-2/9 Signaling Pathway in Neuroblastoma and Glioma
Source: Front Oncol. 2020 Jun 26;10:975. doi: 10.3389/fonc.2020.00975 (PMC7333564; doi:10.3389/fonc.2020.00975)
Supplement: Supplementary file 1 [file Image_1.pdf]

Porf-2 Inhibits Tumor Cell Migration through the MMP-2/9 Signaling Pathway in Neuroblastoma and Glioma

Supplemental

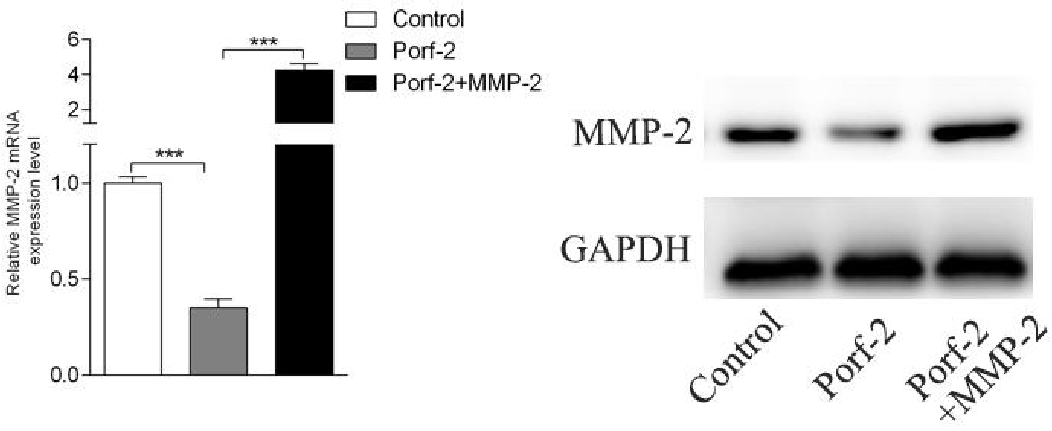

Supplemental Figure 1. Verifying the overexpression of MMP-2 in N-2a. RT-PCR and western blotting confirmed the overexpression of Porf-2. GAPDH was used as a loading control.
