# Supplementary material for: Porf-2 Inhibits Tumor Cell Migration Through the MMP-2/9 Signaling Pathway in Neuroblastoma and Glioma
Source: Front Oncol. 2020 Jun 26;10:975. doi: 10.3389/fonc.2020.00975 (PMC7333564; doi:10.3389/fonc.2020.00975)
Supplement: Supplementary file 2 [file Table_1.DOCX]

| Gene Name | Forward primer | Reverse primer |
| --- | --- | --- |
| MMP-2 | GGGGTCCATTTTCTTCTTCA | CCAGCAAGTAGATGCTGCCT |
| MMP-3 | AGCCTTGGCTGAGTGGTAGA | CGATGATGAACGATGGACAG |
| MMP-7 | GCATTTCCTTGAGGTTGTCC | CACATCAGTGGGAACAGGC |
| MMP-9 | CTGTCGGCTGTGGTTCAGT | AGACGACATAGACGGCATCC |
| CXCR4 | GCATCATCATCTCTAAGC | TAGGATGAGGATGACTGT |
| CXCR6 | AAATCTCCCTCGTAGTGCCC | TGGAACAAAGCTACTGGGCT |
| CXCL4 | CAGCTAAGATCTCCATCGCTTT | AGTCCTGAGCTGCTGCTTCT |
| Ccl4 | GAAACAGCAGGAAGTGGGAG | CATGAAGCTCTGCGTGTCTG |
| CXCR7 | CTACAAACTGCTCAGCACTGAAGG | GCAGTCGCTGCTGTTACATGG |
| ICAM1 | AACAGTTCACCTGCACGGAC | GTCACCGTTGTGATCCCTG |
| VCAM1 | TCGGGCGAAAAATAGTCCTT | CCGGCATATACGAGTGTGAA |
| FAK | CTTGACCCCAACTTGAATCACA | TTCCATACCAGTACCCAGGTG |
| E-cadherin | GGTGTGGGTCAGGAAATCAC | TGTCCCTCCAAATCCGATAC |
| Integrinα1 | TGGCTTCTCACCGTTATCCTA | CACACAAGGCATTGATCTCTCT |
| Integrinβ1 | ATGCCAAATCTTGCGGAGAAT | TTTGCTGCGATTGGTGACATT |
| Integrinβ3 | CAAGAACGAGGATGACTGT | CGGTAGGTGATATTGGTGAA |
| Vinculin | TCTGATCCTCAGTGGTCTGAAC | AAAGCCATTCCTGACCTCAC |
| GAPDH | TGCACCACCAACTGCTTAGC | GGCATGGACTGTGGTCATGAG |
| Porf-2 | CTCACTGCGATGGAACTCTG | GTTGGTTGTCTGCGGGATAG |
| Pannexin1 | GCCAGAGAGTGGAGTTCAAAGA | CATTAGCAGGACGGATTCAGA |
| Pannexin2 | GCGTAGCCTGACACACACTTTA | CACTCACACACATCATCCTCTG |
| NCAM-1 | CGTGGTGGAAGGACACAGT | TGAGCGGAGAATGAGTGACA |
| NCAM-2 | GGAGCATTTGGTTTGGAGTG | GCCAGAGGAGTTTGTCGTATG |
| Claudin1 | TTGATGATGGTTATCGGAACTG | GCTCAGGGAAGATGGTAAGGTA |
| N-cadherin | AATGCTACCTTCCTTGCTTCTG | GAGTTGGGTTCTGGAGTTTCAC |
| Connexin32 | AAACCTTCCCTCCCTTCCTACT | AATAACCTTGAGCCCTGTTGTA |
| Connexin26 | CAAGCCGATTTTGTCTGCAAC | CGATACGGACCTTCTGGGTTT |
| ZO-1 | GCCGCTAAGAGCACAGCAA | GCCCTCCTTTTAACACATCAGA |
| ZO-3 | CTGTGGAGAACGTCACATCTG | CGGGGACGCTTCACTGTAAC |
| Flk-1 | GCG1AGACCATTGAAGTGA | GAAGGAGCCAGAAGAACAT |
| TGF-β1 | GCAACAACGCCATCTATG | CAAGGTAACGCCAGGAAT |
| TIMP-1 | ATCTGGCATCCTCTTGTTG | GTATAAGGTGGTCTCGTTGA |
| TIMP-2 | AGAAGGAGATGGCAAGATG | GGAGGAGATGTAGCAAGG |
| TIMP-3 | CTGCTACTACTTGCCTTGT | TGCTGATGCTCTTGTCTG |

All the primers used in the manuscript
